# Feeding the future: A new potential nutritional impact of *Lactiplantibacillus plantarum* and its promising interventions in future for poultry industry

**DOI:** 10.1016/j.psj.2025.105130

**Published:** 2025-04-03

**Authors:** Muhammad Saeed, Hanan Al-Khalaifah, Afaf Al-Nasser, Tahani Al-Surrayai

**Affiliations:** a College of Agriculture and Biology, Liaocheng University, Liaocheng 252000, China; b Environment and Life Sciences Research Center, Kuwait Institute for Scientific Research, Safat, 13109, Kuwait

**Keywords:** *Lactiplantibacillus plantarum*, Alternatives to antibiotics, Gut microbiota, Encapsulation, Poultry

## Abstract

The increasing demand for sustainable and efficient chicken production has intensified the interest in functional feed additives such as probiotics*. Lactiplantibacillus plantarum* (formerly known as *Lactobacillus plantarum)* is an important probiotic bacterium that has become an essential component in poultry nutrition owing to its diverse advantages. This bacterium improves gut health by regulating the intestinal microbiota, increasing food absorption, and strengthening the immune system. It also alleviates the detrimental impacts of pathogenic bacteria, thereby reducing the reliance on antibiotics and promoting antibiotic-free poultry production. Additionally, *Lactobacillus plantarum* enhances growth performance, feed conversion efficiency, and total flock productivity. Adding Lactobacillus plantarum to chicken feed helps the gut microbiota by encouraging good bacterial communities (e.g., *Eubacterium, Faecalibacterium, Ligilactobacillus, Limosilactobacillus, Blautia* and *Clostridium).* This leads to increased growth in chickens and helps maintain the balance of their gut flora. *Lactobacillus plantarum* has been extensively investigated as a potential feed additive to replace in-feed antibiotics*.* Published literature have revealed that a dietary additive of *Lactobacillus plantarum* improved the health and growth of broilers by improving the balance of bacteria and the metabolism of nutrients in the gut. This study explores the incorporation of *Lactobacillus plantarum* into poultry diets and its importance in sustainable and healthy poultry production. This study will encourage poultry scientists to investigate further before encapsulation. Innovations in *Lactiplantibacillus plantarum,* including its intestine colonization methods and novel strategies to improve its colonization, have the potential to transform the industry. Rapid development of tools and techniques (microencapsulated, nanotechnology, metagenomics, and transcriptome for L. *plantarum*) could help cover research and application shortages.

## Introduction

The prolonged use and widespread misuse of antibiotics lead to critical issues, including diminished animal immunity, increased risk of secondary infections, the presence of antibiotic residues in animal products. These problems pose significant hazards to the safety of animal products and human well-being (Betancur et al., 2020; Parent et al., 2020; Saeed et al., 2023). Historically, feed additives like antibiotics were considered essential for the development of broiler chickens (Khalique et al., 2020; Wu et al., 2024). However, the continued use of antibiotics, on the other hand, subsequently results in the accumulation of microorganisms resistant to several drugs, disruption of gastrointestinal microbial equilibrium, and the presence of pharmaceutical remnants (antibiotic residues) in animal byproducts (He et al., 2022).

To maintain healthy poultry farms and promote faster growth in the birds, there is a need for an alternative to drugs that works well and is safe. Probiotics, which are live bacteria that are not dangerous, have been proven to improve the health of people and animals when administered in adequate amounts (Montoro et al., 2016; Sornplang and Piyadeatsoontorn, 2016). The name “probiotic” is derived from the Greek words *pro,* meaning favor, and *bios,* meaning life (Chauhan et al., 2016).

Probiotics were recently established as a safe and effective dietary approach for enhancing growth and reducing gastrointestinal disorders (Al-Khalaifa et al., 2019; Al-Yami et al., 2022). Probiotics have been shown to boost growth performance and immunity in animals such as yaks, dogs, and mice. This is accomplished by maintaining the balance of the gut microbiota and encouraging the synthesis of organic acids, antagonistic factors, and digesting enzymes to improve the immune system (Gareau et al., 2010; Kim et al., 2021; Wang et al., 2023; 2022).

Among probiotics, *Lactobacillus* species represent a widely used vital category that thrive in many nutrient-rich environments. *Lactobacillus plantarum* belongs to the facultative, heterofermentative species of Lactobacilli. Several studies have focused on the effectiveness of L*. plantarum* when fed to broilers (Hong et al., 2012; Murry et al., 2006). As one of the most adaptable types of lactic acid bacteria (LAB), *Lactobacillus plantarum* is capable of growing in human gastrointestinal tracts that are healthy, as well as in dairy products, meat, and diverse vegetable fermentations*.*(De Vries et al., 2006).

Peng et al. (2016) discovered that providing broilers with nutritional supplements of L. *plantarum* B1 improved their performance and reduced the amount of *E. coli* present in their cecum under normal rearing conditions Yin et al. (2023) found that *Lactobacillus plantarum* GX17 provided multiple benefits for regulating immune in yellow-feathered broilers. Similarly, van den Nieuwboer et al. (2016) noted the high survival rate of *Lactobacillus plantarum* and its ability to modulate the immune system (van den Nieuwboer et al., 2016). Conversely, *Lactobacillus plantarum* was demonstrated to effectively inhibit pathogen invasion (Mohanty et al., 2019). Collectively, these results validate the significant growth and health benefits of *Lactobacillus plantarum*.

A comprehensive understanding of *Lactobacillus plantarum* as a probiotic is essential for creating efficient feed additives and optimizing nutritional management. Millions of bacteria in the gastrointestinal tract of humans and animals play a vital role maintaining homeostasis and overall health (Ghosh et al., 2021). By enhancing intestinal metabolism and gut microbiota balance, *Lactobacillus plantarum* has been shown to enhance the development and health of broiler chickens, indicating the potential of *Lactobacillus plantarum* as a dietary additive to boost broiler chicken growth (Wang et al., 2024). Furthermore, *L. plantarum* JM113 improved gut digestion, absorption, and metabolic processes when challenged with deoxynivalenol (DON) by lowering intestinal barrier injury and boosting the population of beneficial bacteria in broiler chickens (Wu et al., 2018).

Awad et al. (2009) discovered that adding *Lactobacillus* to broiler diets increased villus height and the ratio of the villus height to the crypt depth in the duodenum, while simultaneously decreasing the depth of the ileal crypt (Awad et al., 2009). Several other studies have demonstrated that due to their ability to stimulate growth, multi-strain Lactobacillus supplements have the potential to be utilized as probiotics in industrial chicken production (Gheorghe et al., 2018; Lokapirnasari et al., 2020). Qiao et al. (2019) demonstrated how L. *plantarum* might modify metabolic processes and nutrition use by controlling the gut microbiota Of the broilers. Another study verified that broiler productivity, immunological response, and intestinal microecological balance increased when L. *plantarum* was added as a feed supplement (Shen et al., 2014).

Numerous studies have shown that supplementing animal feed with *Lactobacillus plantarum* can help restore a healthy microbiome in the intestines, increase the feed conversion efficiency, enhance the immune system, and facilitate the digestion and assimilation of nutrients, all of which contribute to the growth of livestock and poultry (Blajman et al., 2017; De Souza et al., 2018). L. *plantarum* has garnered significant interest from farmers due to its potential as a drug-resistant, residue-free, and environmentally benign microbial additive. It is classified within the genus of lactic acid bacteria (LAB) (Stiles and Holzapfel, 1997).

In comparison with other LAB, L. *plantarum* is capable of synthesizing distinctive lactic acid bacteriocins throughout its reproductive phase (Pieper et al., 2009; Zeng et al., 2021; Additives et al., 2017; Pieper et al., 2009). Furthermore, L. *plantarum* is widely used in both human and animal feed and is considered one of the safest species (Kanmani et al., 2013).

The aforementioned literature indicates that *Lactiplantibacillus plantarum* is one of the best possible substitutes for antibiotic growth promoters (AGP). It enhances intestine histomorphology, immunological response, nutritional digestibility, growth performance, and meat quality in poultry. Nevertheless, the use of *L. plantarum* in chicken diets remains relatively unexplored, and little research has been done on the effects of its fermentation products on various poultry species.

### Bioactivities of *Lactiplantibacillus plantarum*

**Fig. 1:**
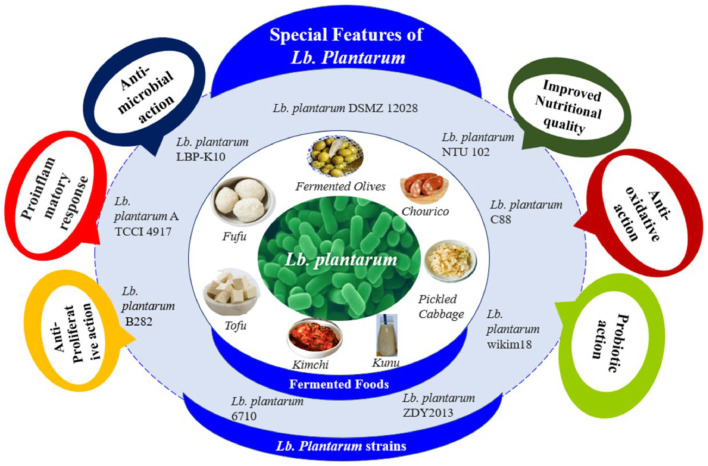
It is showing the different benefits of Lb. *plantarum* and its different strains (Yilmaz et al., 2022).

The probiotic, antimicrobial, indigenous gut modulation, antioxidant, and immunomodulatory properties of *Lactiplantibacillus plantarum* render them a valuable element in poultry production. *L. plantarum* strains can live in different environments, such as the human gut, vegetables, meat, fish, dairy, and other fermented foods (Yilmaz et al., 2022) (Fig. 1).

#### Possible roles and probiotic properties of *Lactiplantibacillus plantarum*

Literature indicates that *L. plantarum* strains possess numerous functional qualities, including enhancement of nutritional quality and taste, antioxidant and antibacterial effects, and extension of food shelf life, and the reduction of unwanted chemicals (Yilmaz et al., 2022). The European Food Safety Authorities (EFSA) has granted *L. plantarum* a Qualified Presumption of Safety (QPS) status, which means that it is safe for consumption. Similarly, the United States Food and Drug Administration (US FDA) has classified *L. plantarum* as "Generally Recognized as Safe" (GRAS) (Hazards et al., 2017).

*L. plantarum*, a prominent member of the *lactobacilli genus*, is widely used as a probiotic due to its beneficial features, including antioxidants, GI tolerance, adhesion, and antibacterial activities. Although previous research concentrated on isolating probiotic strains and bioactive metabolites of *L. plantarum*, current research explores their application in food industry and their adaptation to environmental stress (Huang et al., 2021; Liu et al., 2022a; Patil et al., 2020). Recent studies have highlighted the probiotic potential of *L. plantarum* strains isolated from fermented foods (Pan et al., 2021).The biological and probiotic qualities of *Lactiplantibacillus plantarum* strains taken from fermented foods are summarized in Table 1.

**Table 1:** The biological and probiotic qualities of *Lactiplantibacillus plantarum’* strains that were taken from fermented foods.

Foods that ferment	Isolated L. *plantarum* strains	Probiotic activities	Refs.
Pickles food	Wikim0112 and KACC11451	Excellent intestinal epithelial adhesion (60-62 %) and pathogen inhibition. High antioxidant ability (more than 70 % activity, comparable to superoxide dismutase)	(Jeong et al., 2021)
Kimchi food	L. *plantarum* LRCC5314	This strain is very constant and can live in bile acid (0.2 %) and low pH (=2.0). It sticks to Caco-2 cells 89.9 % of the time.	(Yoon et al., 2021)
Dhokla batter food	*Lactiplantibacillus plantarum’* DKL3 and JGR2	It is capable of effectively absorbing acid and bile and adhering to the epithelial cells of the intestine. It also produces exopolysaccharides, folate, and riboflavin.	(Surve et al., 2022)
Kimchi food	*Lactiplantibacillus plantarum’* strains	LRCC5193 of L. *plantarum* demonstrated high-stress tolerance.	(Lim et al., 2018)
Fermented Paocai	*Lactiplantibacillus plantarum’* DMDL 9010	Good tolerance in the digestive tract, as well as antioxidant and antibacterial effects.	(Liu et al., 2022a)
Traditional cheese from Algeria called "Jben"	SJ14	Good resistance to bile and acid, good intestinal cell adhesion, antibacterial activity against opportunistic and pathogenic microbes, and antifungal activity.	(Metrouh et al., 2022)
Theobroma cacao	*Lactiplantibacillus plantarum’* Lp03, Lp289, and Lp291	Every strain was heat-tolerant, low-pH, antibacterial, and generated hydrogen peroxide.	(das Neves Selis et al., 2021)

#### Antimicrobial activity of *Lactiplantibacillus plantarum*

According to research, L. *plantarum* is capable of producing a wide range of antimicrobial chemicals, including bacteriocin, hydrogen peroxide, and acids (Siamansouri et al., 2013). Numerous studies have reported that during its fermentative metabolism, *L. plantarum* generates many antimicrobial chemicals (in addition to bacteriocins). These may organic acids such as lactic, citric, isobutyric, and acetic acids, as well as ethanol, diacetyl, and H_2_O (Liu et al., 2022b; Markkinen et al., 2022).

One study examined the antibacterial activity of *Lactiplantibacillus plantarum* strains APsulloc 331261 and APsulloc 331266. The findings showed that these strains of *L. plantarum* may enhance the balance of the skin microbiota by suppressing skin pathogenic strains (Chae et al., 2021). The production of bacteriocins and the inhibitory action of the pig-isolated *L. plantarum* LP5 strain against the STEC EDL933 strain indicate its potential as a probiotic (Ruiz et al., 2023). Three distinct strains of Lb. *plantarum*—LP1, LP2, and LP3—demonstrated excellent antibacterial activity against *Escherichia coli* ATCC 25922 and *Staphylococcus aureus* ATCC 25923 (Arena et al., 2016; Wang et al., 2018). Additionally, certain studies have reported the antibacterial activity of *Lactiplantibacillus plantarum* strains against pathogenic microorganisms found in food.

Based on these results, *L. plantarum* should be promoted as a possible bio-control method against pathogenic microorganisms in both food and feed industries. *L plantarum* is not only a more environmentally friendly choice (it can be used instead of artificial antibacterials) but it also holds significant potential for the making of functional foods.

#### Antioxidant activity of *Lactiplantibacillus plantarum*

Given the significance of oxidation processes, many studies have evaluated the antioxidant ability of *L. plantarum,* with favorable results. Using the 2,2-diphenyl-1-picrylhydrazyl (DPPH) assay and the superoxide anion (O2-) scavenging activity method, several researchers have shown that *L. plantarum* DMDL 9010 possesses excellent antioxidant capacity, with the supernatants exhibiting a higher antioxidant capacity compared to the bacterial precipitates (Liu et al., 2022a, b; Shu et al., 2024; Wang et al., 2017).

In contrast, Tian et al. (2022) discovered that, using the DPPH assay and reducing power, the extracts derived from cell-free L. plantarum N-1 showed lower antioxidant ability than those with bacterial cells. This implies that the intact cells of L. *plantarum* N-1 contain antioxidant chemicals. Despite this difference, both extracts demonstrated impressive antioxidant potential, surpassing that of other probiotic strains.

Furthermore, Jeong et al. (2021) demonstrated that L. *plantarum* Wikim0112 and KACC11451 exhibited over 70 % activity comparable to that of superoxide dismutase (SOD). Using the DPPH and ABTS assays, the Wikim0112 strain was exhibited to have the highest antioxidant capacity. Similarly, *L. plantarum* KU15149 showed a high concentration of antioxidants, as measured by DPPH assay and β-carotene bleaching inhibition (Han et al., 2020; Lee et al., 2008; Park et al., 2014).

Using the cellular antioxidant activity (CAA) tests, Tang et al. (2018) discovered that *L. plantarum* MA2 has a high antioxidant capacity in the cell-free extract of the logarithmic phase, compared to the stationary phase and the fermentation supernatant. The authors revealed that intracellular antioxidant enzymes—such as catalase, feruloyl esterase, glutathione peroxidase, glutathione reductase, glutathione transferase, NADH oxidase, and superoxide dismutase—and non-enzymatic compounds were shown to be responsible for the antioxidant capacity of cell-free extract (Lin et al., 2020; Mu et al., 2018).

#### Resistance to gastrointestinal activity of *Lactiplantibacillus plantarum*

Recent research on *Lactiplantibacillus plantarum* PMO08, isolated from kimchi, have shown that it may improve cholesterol metabolism, immunological response, and gastrointestinal health (Oh et al., 2022). In this adverse environment, various strains of *L. plantarum* have demonstrated probiotic potential owing to their tolerance to pH and bile. This resilience is achieved through strategies including the preservation of intracellular pH homeostasis, swift recycling of damaged proteins, and activation of multiple stress response pathways, induction of bile salt hydrolase, and the maintenance of the proton motive force (Fidanza et al., 2021). The *L. plantarum* MA2 and B23 strains demonstrated significant tolerance and were capable of enduring pH levels as low as 2.5–3 (Tang et al., 2018).

Nath et al. (2020) similarly demonstrated that *L. plantarum* GCC_19M1 exhibited considerable tolerance to low pH environments, with survival rates ranging between 93.48 % and 96.97 % when subjected to simulated gastric juice (pH=3). Furthermore, L. *plantarum* GCC_19M1 showed significant resistance to 0.3 % bile, 0.5 % pancreatin, and 5 % NaCl. L. *plantarum* SJ14 showed notable resistance to conditions of the human gastrointestinal system that resembled bile-like acidity. Strains of *L. plantarum* isolated from pickles and kimchi (KACC11451 and Wikim0112, respectively) showed extraordinary resistance to gastrointestinal environment (Liu et al., 2022a; Metrouh et al., 2022).

Supplementation with *Lactiplantibacillus plantarum* P-8 may promote growth and reduce stress in weaned piglets without antibiotics by changing their gut flora. This supplementation significantly decreased ADFI, FCR, and ileal mucosal crypt depth while boosting villus height-to-crypt depth ratio, hepatic glutathione peroxidase and catalase activity, and serum interleukin. Antibiotics and probiotics affected the gut microbiomes of the piglets (Yu et al., 2024).

*L. plantarum* MCC5231 was found to produce exopolysaccharides and biofilms, which may protect the gut. These data suggest that *L. plantarum* MCC5231 is a safe probiotic option for food and feed industries (Goel and Halami, 2024).

#### Anti-inflammatory activity of *Lactiplantibacillus plantarum*

*L. plantarum* A7 exhibited an anti-inflammatory effect by altering the distribution of CD4 + T-cell subsets, hence facilitating Th2 differentiation and suppressing Th1 differentiation (Salehipour et al., 2017). Similarly, *Lactiplantibacillus plantarum* IDCC 3501, isolated from kimchi, was tested for safety and anti-inflammatory efficacy. LPS-induced RAW 264.7 macrophages treated with L. *plantarum* IDCC 3501 cell-free supernatant resulted in considerable reduction in the mRNA expression of inflammatory markers (TNF-α, IL-1β, and IL-6) (Yang et al., 2021).

Another study thoroughly investigated the anti-inflammatory and antioxidant properties of *Lactiplantibacillus plantarum* A106, isolated from traditional Chinese pickles, on LPS-induced RAW264.7 macrophages. These findings indicate that *L. plantarum* A106 regulates inflammatory and apoptosis-related gene expression, thereby restoring mitochondrial membrane potential and enhancing antioxidant activity (Qin et al., 2024). Probiotics (*Lactiplantibacillus plantarum*) have demonstrated anti-inflammatory properties by obstructing the activation of MAPKs and NF-κB (Cristofori et al., 2021). Although several studies have illustrated the advantageous benefits of probiotics and their capacity to influence the immune system, hence offering anti-inflammatory properties, the underlying mechanisms of action of these bacteria remains largely unexplored (Vincenzi et al., 2021). Consequently, all potential processes and innovative approaches for inflammation reduction are of significant interest.

In conclusion, *L. plantarum* Z22, isolated from naturally fermented vegetables, has anti-inflammatory properties that holds potential to prevent and treat inflammatory diseases. It can also be used to develop nutritional supplements to meet the demand for anti-inflammation and immune enhancement (Wang et al., 2024). Animal studies and clinical trials have indicated the health benefits associated with *L. plantarum* supplementation, especially in relation to gastrointestinal and inflammatory conditions (Fidanza et al., 2021). Future research must examine its *in vivo* safety and efficacy to ensure its continued use.

#### Immunomodulatory activity of *Lactiplantibacillus plantarum*

*L. plantarum* has demonstrated significant immunomodulatory effects through the regulation of gut microbiota, potentially reducing the risk of many diseases (Bu et al., 2023; Zhao et al., 2023). The effectiveness of the immune system is based on the reliable transmission of specific, functioning proteins. *L. plantarum* has an exceptional capacity to elicit immunological responses, according to an examination of its functional genes (Liu et al., 2015; Zhang et al., 2019). Certain probiotic strains of *L. plantarum* may serve as effective immunomodulators that modulate the Th1/Th2 balance and elicit advantageous immune responses (Kawashima et al., 2011; Smelt et al., 2012). One such strain is L. *plantarum,* which aids in the prevention of Th2-associated immunological disorders.

*In vitro* studies have proven that the production of pivotal polarized cytokines, IL-12 and IL-10, by dendritic cells (DCs) was augmented in the presence of L. *plantarum* NCIMB8826, thereby skewing the T-cell response towards advantageous Th1 profiles (Hisbergues et al., 2007; Pochard et al., 2005; Rigaux et al., 2009). In another study, supplementation of Aflatoxin B1 diet with *Lactiplantibacillus plantarum* significantly reduced immune-inflammatory gene expression (Nrf2, IL-10, and BCL-2) and apoptotic gene CASP3. This study suggests that *L. plantarum* (1 × 109 cfu/g/kg diet) may protect rabbits from Aflatoxin B1 by boosting antioxidant and immunological activity, while lowering apoptosis and inflammation (Saghir et al., 2024).

Supplementing poultry diet with probiotics may produce the following effects: (1) changing the gut microbiome; (2) strengthening the immune system in chickens (Jha et al., 2020). *Bacillus* probiotics have gained increasing attention as an alternative to antibiotics growth promoters (AGPs) due to their health-enhancing properties and resilience to the rigorous industrial conditions of poultry feed production (Grant et al., 2018). When used as feed supplements, probiotics facilitate optimal feed digestion, thereby enhancing nutrient availability for accelerated growth. In addition to enhancing immunity in poultry, probiotics enhance the qualitative characteristics of meat and eggs (Alagawany et al., 2018). One study shows that L. *chiayiensis* AACE3 is a great feed additive that can serve as an alternative to aureomycin. It also helps chickens grow healthy during the brooding period by positively influencing their gut microbiome (Kang et al., 2023).

### Antibacterial activity of *Lactiplantibacillus plantarum*

*Lactobacillus plantarum* demonstrated inhibitory effects against several pathogenic bacteria, including *Salmonella typhimurium, Listeria monocytogenes, E-coli*, and vancomycin-resistant *Enterococci* (Chen et al., 2017; Zang et al., 2024). Enzymes, proteins, peptides, fatty acids, and bacteriocins, exemplify postbiotic entities with antibacterial properties. Additional examples include organic acids and hydrogen peroxide (Aghebati-Maleki et al., 2021). Bacteriocins, in particular, are ribosomally generated antimicrobial peptides that suppress or eliminate bacterial strains, both closely related or unrelated, without harming the producing bacteria due to the presence of self-defensive proteins (Vogel et al., 1993; Yang et al., 2014).

Microbe fermentation produces cell-free supernatants (CFS), which contain microbial metabolites and nutrients from the growth medium they did not ingest. Lactobacillus CFS may exhibit antibacterial properties due to the presence of fatty acids, proteinaceous polymers, and organic acids. CFS from Lactobacillus is antimicrobial due to lactic acid, acetic acid, and other compounds (Mani‐López et al., 2022). Bacteriocins present in these supernatants offer multiple benefits, such as inhibiting the emergence and proliferation of pathogenic bacteria, spore formation, and the creation of pores in the cell membranes of pathogens (O'Connor et al., 2020; Wu et al., 2021; Xu et al., 2023).

**Fig. 2:**
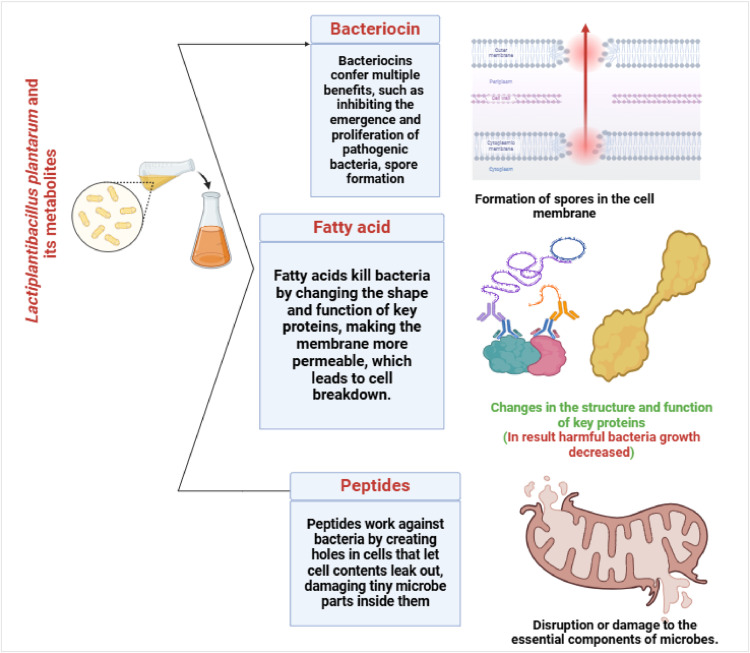
Certain metabolites of *Lactiplantibacillus plantarum* work against bacteria in the gut, stopping the bacteria from dividing (Antibacterial activity).

Fatty acids can inhibit the growth of bacteria through several mechanisms, including the modification of the morphology and function of sensitive components such as proteins, the enhancement of membrane permeability and subsequent cell lysis, the disintegration of the electron transport chain, the interference with the structure as well as the function of enzymes, and the disruption of the electron transport chain (Ababouch et al., 1992; Yoon et al., 2018). Peptides work against bacteria by creating pores in cells, causing leakage of cell contents, damaging tiny microbe parts inside them, triggering potentially fatal processes such as hydrolase production that damage cell walls, and increasing the acidity of the cell membrane of bacteria (Isaac-Bamgboye et al., 2024; Zasloff, 2002). Certain metabolites of *Lactiplantibacillus plantarum* also work against bacteria in the gut, inhibiting division of the bacteria (Fig. 2).

#### Effects of *Lactiplantibacillus plantarum* supplementation on poultry production

**Fig 3:**
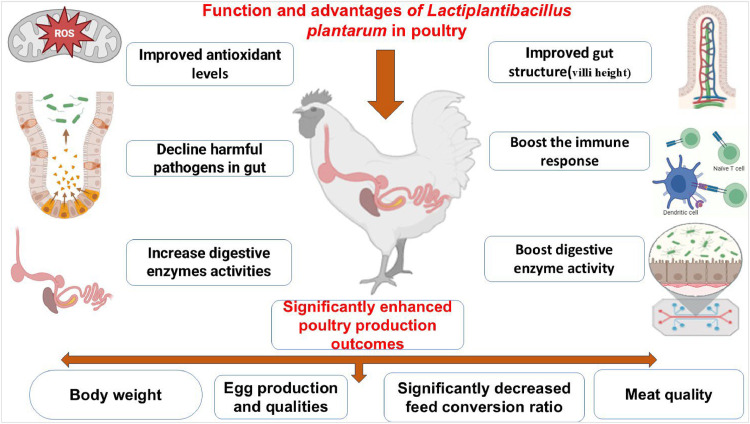
A beneficial benefits of *Lactobacillus plantarum* in poultry.

Since antibiotics are banned as growth promoters, many probiotics have been developed as alternatives. Numerous studies have shown that *Lactiplantibacillus plantarum* enhances intestinal health; however, further research is needed to determine its in replacing antibiotics as a broiler feed growth booster. *L. plantarum* has potential as an alternative to antibiotics in poultry nutrition (Salim et al., 2013; Zhang et al., 2016). Alongside *Lactobacillus* spp., many species of *Enterococcus* spp. have been recognized as effective probiotics for poultry. For instance, the *Enterococcus faecium* boosts blood parameters and improves hatchability, growth performance, and intestinal morphology in broilers (Wu et al., 2019). *Lactobacillus plantarum* may have numerous effects on poultry health and production, as illustrated in Fig. 3.

#### Effects on broiler growth performance and meat quality

Broiler chicken diets supplemented with a *Lactobacillus plantarum* metabolite has been proven to lower cholesterol levels in the plasma and breast meat (Loh et al., 2013). The research study sought to examine the effects of *Lactiplantibacillus plantarum* (L. *plantarum*) on growth performance, oxidative resistance, immunity, and cecal microbiota in broilers. The results indicated that *L. plantarum* supplementation significantly enhanced average daily weight gain (ADWG) (*p* < 0.05) and decreased the feed-to-gain ratio throughout the supplementation period (*p* < 0.05). The best *L. plantarum* dosage depends on the strain, dosing technique, and desired results. Supplementation with *L.* plantarum HJLP-1 at an amount of 5 × 10⁸ CFU/kg of feed enhanced average daily weight gain and feed conversion ratios in broilers over a 42–day period (Yang et al., 2024). Similarly, supplementation of at 2.6 × 10⁹ CFU/g of feed, enhanced growth, meat quality, and intestinal health in broilers over six weeks (Liu et al., 2023). Research suggests that dosages of *L. plantarum* for poultry nutrition should range from 5 × 10⁴ to 2.6 × 10⁹ CFU per kilogram of feed per chicken. However, strain, delivery route, and production goals must be considered while selecting dosage.

Feeding L. *plantarum* to broiler chickens has the potential to enhance growth performance, support gut microbial balance, help prevent pathogen infections, and mitigate intestinal barrier damage caused by toxins (Markkinen et al., 2022; Wu et al., 2018; Zeng et al., 2018). *Lactoplantibacillus plantarum* has been demonstrated to support growth in broiler (Deepthi et al., 2017; Gao et al., 2017; Peng et al., 2016).

A study investigating the effects of *Lactiplantibacillus plantarum* on oxidative stress and NLRP3 inflammasome activation in broiler breast meat found that adding *Lactiplantibacillus plantarum* P8 to the diet of oxidatively stressed broilers reduced oxidative stress, enhanced mitophagy, and inhibited NLRP3 inflammasome activation (Yuan et al., 2023). The supplementation of L. *plantarum* postbiotic can serve as an effective alternative to antibiotic growth promoters as it enhances growth performance, mucin production, tight junction permeability, and immunological status in broiler chickens by improving gut health and promoting beneficial bacterial colonization (Chang et al., 2022).

Three probiotic strains—*Lacticaseibacillus rhamnosus* AG16, *Limosilactobacillus fermentum* HFD1, and *Lactiplantibacillus plantarum* LS-4.4—have been proven to have an impact on the quality of quail meat. All three strains reduced breast meat water retention capacity and cooking loss relative to the control group in quail. Probiotic *Lactobacilli* made quail flesh less stiff, thereby reducing the chewing and cutting effort (Tsyganov et al., 2023). Additionally, there has been a drop in cholesteryl ester and a rise in very low-density lipoprotein particles. The ability to deconjugate bile salts also gets better by increasing the amount of *Lactobacillus* in the small intestine digesta. The cholesterol-lowering effect observed in chicken meat can be linked to the growth of microfloral *Lactobacillus,* which accelerates bile acid catabolism of cholesterol. Also, administration of postbiotic L. *Plantarum* RG14, *L. plantarum* RI11, and inulin in broiler chicken resulted in lower drip loss and enhanced breast muscle lightness (Kareem et al., 2015).

#### Effect on egg production

**Fig. 4:**
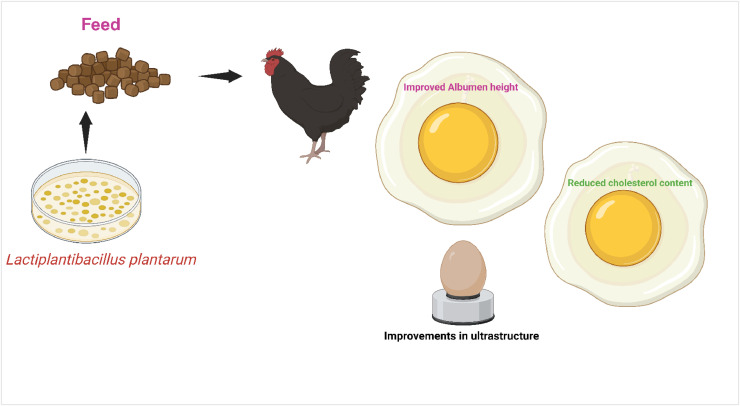
Promising bioactivities of *Lactiplantibacillus plantarum* on egg qualities.

Supplementation with *Lactiplantibacillus plantarum* 18 resulted in higher laying rates (*p* < 0.05) and enhanced egg quality, including shape index, albumen height, Haugh unit, and eggshell strength (*p* < 0.05), as along with notable increase in ultrastructure (Fig. 4). The findings shed light on the genomics of LP18 and the genes responsible for its survival and colonization in the gut. This study shows that LP18 holds potential as a probiotic to improve poultry productivity, egg quality, and lipid metabolism (Zhang et al., 2023).

The three strains of *L. plantarum* (COM456) that produced 0.6 % liquid metabolite combinations were RI11, RG11, and RG14, which demonstrated the most significant improvement in the egg production of the hens (Choe et al., 2012). After adding metabolites to the meal, there was a marked reduction in the amount of cholesterol found in the egg yolks. The reason behind this reduction is the ability of *Lactobacillus*, an intestinal bacterium, to deconjugate bile salts, thereby reducing the cholesterol available for integration into lipoproteins.

Oocyte vitellogenesis receptors allow very low-density lipoproteins to transport blood cholesterol across ovarian membranes and deposit it into the developing yolk (Elkin and Lorenz, 2009). According to an experiment on the effects of supplementing diet of laying hens with postbiotic COM456 at 0.6 %, the metabolite combinations led to an increase in hen-day egg output and egg mass (Lee et al., 2009).

#### Effect on intestinal barrier integrity and gut pathogens

Chickens have a large microbial population in their gastrointestinal tract (GIT), which helps with nutrition absorption, immunology, and disease resistance (1). Changes in the GIT microbiota can affect feed efficiency, production, and health of poultry (Jeurissen et al., 2002). Postbiotics function by boosting LAB populations and improving intestinal shape, thereby reducing the risk of villi damage caused by gut pathogens (Svetoch et al., 2011).

The probiotic *Lactiplantibacillus plantarum* (LP) is widely used in poultry production. Recent research has indicated that its postbiotics—comprising bacterial components and metabolites—improve nutrition intake, immunological response, and gut microbiota, thus promoting poultry product growth and quality (Chang et al., 2022; Danladi et al., 2022). *Lactobacillus* has demonstrated beneficial impacts on broiler performance, modification of the gut microbiome, and suppression of infections through multiple mechanisms, including competitive exclusion, organic acid generation, and synthesis of antimicrobial compounds (Krysiak et al., 2021; Neal-McKinney et al., 2012). Supplementation with L. *plantarum* has been shown to modify the composition of gut microbiota composition by enhancing beneficial bacteria and reducing dangerous strains. This modulation fosters a healthy microbial equilibrium, crucial for optimal gastrointestinal function and defense against infections (Hao et al., 2024). These various mechanisms show that *L. plantarum* could be used as a probiotic to improve the integrity of the intestinal barrier and protect against gut pathogens, which would improve overall gut health.

The probiotic potential and antibacterial activities of *Lactiplantibacillus plantarum* were examined against *Salmonella typhimurium*, and *Escherichia coli*(O157:H7). The findings showed that *B. longum* and *L. plantarum* have probiotic potential for managing *E. coli* O157:H7 in poultry (Igbafe et al., 2020). *Lactobacillus* species can produce and secrete antimicrobial chemicals such as Bacteriocins and volatile fatty acids (VFAs). These substances create an unfavorable environment for the growth of dangerous bacteria such as *Salmonella* and *E. coli*. The VFAs reduce the pH and inhibit the viability of *Enterobacteriaceae,* promoting gut health (Reid, 2001). Furthermore, the reduction of pathogens in intestine by metabolite combination increases the ability of *Lactobacillus* to colonize the gut microbiota through competitive exclusion.

Another factor that reduces these *Enterobacteriaceae* is the bacteriostatic effects of VFA in caeca (van der Wielen et al., 2000). The antimicrobial effects of postbiotics are primarily attributed to bacteriocins and SCFAs, as per (Aguilar-Toalá et al., 2018). Maltol also is a promising postbiotic substitute for antibiotics for enhancing the growth and immunity of poultry that exhibit gastrointestinal illnesses (Park et al., 2021). The expansion of *Lactobacillus* populations while diminishing the prevalence of gram-negative bacteria (Eddula, 2022). The postbiotics derived from L. *Lactiplantibacillus plantarum* increase the growth and intestinal health of chickens by reducing the number of harmful microorganisms in their intestine, leading to better performance.

**Fig. 5:**
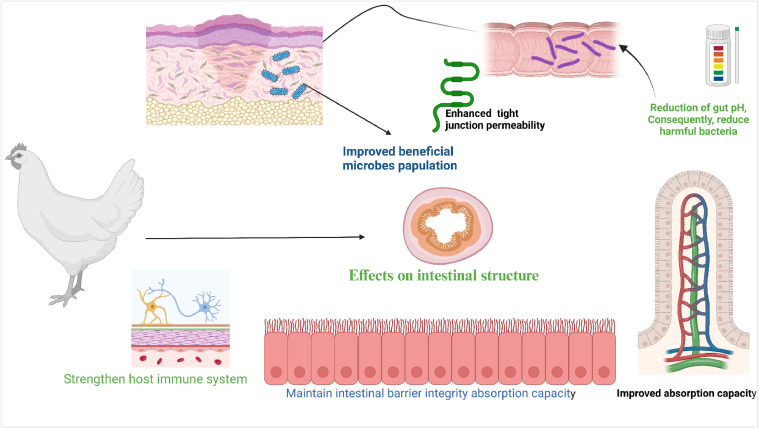
The potential effects of *Lactiplantibacillus plantarum* on intestinal barrier integrity and gut pathogens in chicken.

Published literature has reported that antimicrobial metabolites in L. *plantarum*, including organic acids and bacteriocins, can increase tight junction permeability, decrease gut pH, and inhibit the growth of opportunistic infections in poultry. These metabolites can support colonization of beneficial bacteria and improve overall gut health and gastrointestinal function of poultry (Fig. 5). Consequently, they influence the local environment or modify the gene expression of the host. The effects of *Lactiplantibacillus plantarum* on poultry health and performance are summarized in Table 2.

**Table 2:** Effects of *lactiplantibacillus plantarum* on poultry health and performance.

*lactiplantibacillus plantarum's strain*	Species	Bioactivities	Refs.
*Lactiplantibacillus plantarum* RG11 and its strains *(*RG14, RS5 and RI11)	Broilers	RI11 could reduce *Salmonella*, ENT, and *E. coli* numbers in chicken and be an alternative antibiotic growth booster and anti-stress medication.	(Humam et al., 2019)
*Lactiplantibacillus plantarum* RS5	Layer birds	*Lactiplantibacillus plantarum* RS5 enhanced growth and reduce heat stress. It greatly enhanced egg production	(Farran et al., 2024)
*Lactiplantibacillus plantarum*	Layer birds	Significantly increased in small intestine villi height	(Choe et al., 2012)
*Lactiplantibacillus acidophilus*	Broilers	Reduction in Coliform count	(Abd El-Ghany et al., 2022)
*Lactiplantibacillus animalis*	Quails	It substantially reduced *E. coli* and it is as feed additives that may replace antibiotic growth boosters in quail diet.	(Kareem, 2020)
*Lactiplantibacillus plantarum*	Broilers	Increased in villi height in duodenum, jejunum and ileum and demonstrated that 0.2 % is the optimal broiler food amount to substitute antibiotic growth boosters.	(Loh et al., 2010)
*Lactiplantibacillus plantarum*	Broiler chicks	*Lactoplantibacillus plantarum* 0.1 % supplement improved growth performance, mucin synthesis, immunological status, tight junction permeability, gut health.	(Chang et al., 2022)
*L. plantarum* RG14 and RI11	Broilers chicken	Adding *Lactiplantibacillus* strains made a big difference. The number of Enterobacteriaceae in broilers dropped.	(Kareem et al., 2016)
*Lactiplantibacillus plantarum*	Broilers chicken	The incorporation of a metabolite combination supplement also enhanced the population of faecal lactic acid bacteria, increased intestine villus height.	(Thanh et al., 2009)
*Lactiplantibacillus plantarum*	Broilers chicken	*Lactoplantibacillus plantarum* had the reduction of harmful microbes while enhancing helpful microbes*.*	(Danladi et al., 2022)
*Lactiplantibacillus plantarum*	Broilers chicken	The observed favorable alteration of the microbiota is particularly significant, as the incorporation of these alternative nutrients may enhance the gut health of broilers.	(Ferrocino et al., 2023)
*Lactiplantibacillus plantarum FRT4*	laying hens	*Lactiplantibacillus* plantarum (Lp. *plantarum*) boosted laying rate and reduced liver lipid buildup.	(Li et al., 2024)

#### A promising new potential interventions in future

By delving further into *Lactiplantibacillus plantarum*, scientists will be able to better understand its mechanisms for combating pathobionts and develop novel pharmabiotics and pharmacological methods that are less invasive and produce more targeted physiological effects (Tomar et al., 2015). Although many alternative approaches have shown favorable effects, they are often considered as lacking consistency and producing variable results from farm to farm. Additionally, their operation is yet to be sufficiently explained. As antibiotic use in poultry industry is being reduced, maximizing performance and preserving animal output will depend on identifying the optimum combinations of various alternatives and proper management and husbandry practices (Gadde et al., 2017).

The potential benefits of *Lactiplantibacillus plantarum* range from improved chicken productivity, gut health, meat quality, and immunity make them essential for safe and sustainable poultry farming worldwide. If their specific characterization and mechanism of action are better understood, these biomolecules will be highly useful in poultry. This requires extensive research on these compounds to prove their health benefits in poultry, especially given the growing danger of AMR worldwide. The effectiveness of *Lactiplantibacillus plantarum* from various sources needs to be demonstrated in several disease models, such as colibacillosis, necrotic enteritis, and coccidiosis, which can affect the financial viability of poultry farming.

**Fig. 6:**
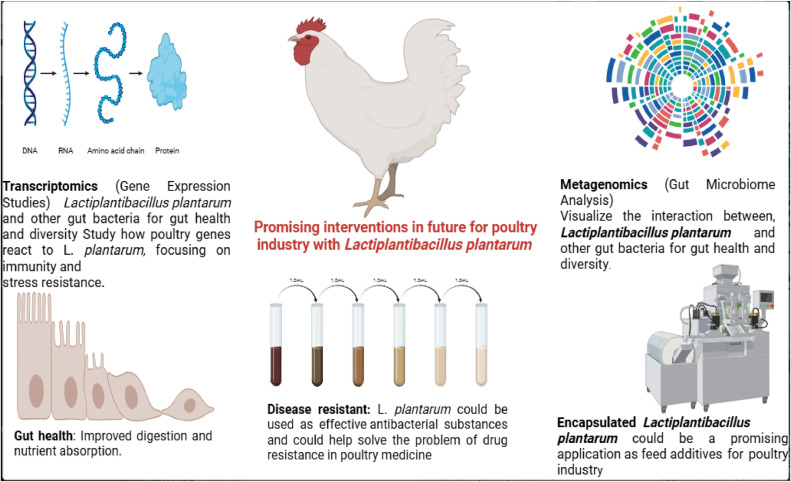
*Lactiplantibacillus plantarum* supports the poultry industry through tools like metagenomics and transcriptomics and encapsulation. It highlights the key benefits, such as improved gut health, disease resistance, and growth.

*Lactiplantibacillus plantarum* may help reduce heat-stress in fast-growing broilers and improve their respiratory health due to its antioxidant properties. Most investigations focus on *Lactiplantibacillus plantarum* as native AGP replacements, and nanotechnology is expected to boost their cellular bioavailability. Microencapsulation protects *Lactiplantibacillus plantarum* against proventriculus and gut environmental hazards such acidity, bile salts, chemicals, antimicrobials, active oxygen (for anaerobic bacteria). The application of *Lactiplantibacillus plantarum* in poultry is predicted to expand significantly with more preclinical trials by using metagenomics and transcriptomics, which will help understand how stresses affect chicken signaling networks. Microencapsulation has been suggested as one of the most effective approaches as shown in Fig. 6.

## Conclusion

Since antibiotics are banned as growth promoters, many probiotics have been developed as alternatives. *Lactiplantibacillus plantarum* (L. *plantarum*), a prevalent strain of LAB, has demonstrated probiotic properties. In the past, *L. plantarum* has demonstrated beneficial benefits on broiler performance, gut microbiota modulation, pathogen suppression, anti-heat stressor properties, making it a promising candidate as an antibiotic substitute for major commercial applications in the chicken industry. A number of harmful bacteria, such as *Salmonella typhimurium, Escherichia coli*, and *vancomycin-resistant enterococci*, are inhibited by *Lactobacillus plantarum*.

The aforementioned studies have demonstrated the potential of *Lactiplantibacillus plantarum* (*L. plantarum*) as feed additives to enhance the antioxidant content and meat quality of poultry. These findings highlight the necessity to promote *L. plantarum* as an effective biocontrol tool in the feed industry. *L. plantarum* provides a sustainable alternative to synthetic antibacterial agents and has considerable potential in chicken feed production. *L. plantarum* strains can be efficient antibacterials and help address poultry medication resistance. However, further research is required to evaluate if *Lactiplantibacillus plantarum* can fully replace antibiotics as a broiler feed growth booster, and to determine its mode of action and optimal poultry husbandry dosage. The encapsulation of *Lactiplantibacillus plantarum* as poultry feed additive represents a promising strategy in poultry industry.

## Disclosures

The authors say they don't have any known personal or financial relationships or financial interests that could have seemed to affect the work in this study.

## Declaration of competing interest

The authors declare that they have no conflicts of interests.
